# Stiefel Manifold Dynamical Systems for Tracking Representational Drift

**DOI:** 10.64898/2026.03.07.710319

**Published:** 2026-03-10

**Authors:** Hyun Dong Lee, Aditi Jha, Stephen E. Clarke, Michael P. Silvernagel, Paul Nuyujukian, Scott W. Linderman

**Affiliations:** 1 Computer Science Department, Stanford University; 2 Wu Tsai Neurosciences Institute, Stanford University; 3 Department of Statistics, Stanford University; 4 Bioengineering Department, Stanford University; 5 Neurosurgery Department, Stanford University; 6 Electrical Engineering Department, Stanford University

## Abstract

Understanding neural dynamics is crucial for uncovering how the brain processes information and controls behavior. Linear dynamical systems (LDS) are widely used for modeling neural data due to their simplicity and effectiveness in capturing latent dynamics. However, LDS assumes a stable mapping from the latent states to neural activity, limiting its ability to capture representational drift—gradual changes in the brain’s representation of the external world. To address this, we introduce the Stiefel Manifold Dynamical System (SMDS), a new class of model designed to account for drift in neural representations across trials. In SMDS, emission matrices are constrained to be orthonormal and evolve smoothly over trials on the Stiefel manifold—the space of all orthonormal matrices—while the dynamics parameters are shared. This formulation allows SMDS to leverage data across trials while accounting for non-stationarity, thus capturing the underlying neural dynamics more accurately compared to an LDS. We apply SMDS to both simulated datasets and neural recordings across species. Our results consistently show that SMDS outperforms LDS in terms of log-likelihood and requires fewer latent dimensions to capture the same activity. Moreover, SMDS provides a powerful framework for quantifying and interpreting representational drift. It reveals a gradual drift over the course of minutes in the neural recordings and uncovers varying drift rates across dimensions, with slower drift in behaviorally and neurally significant dimensions.

## 1 Introduction

Latent dynamical systems are widely used in systems neuroscience to understand the evolution of neural activity over time (Smith and Brown, 2003; Eden et al., 2004; Macke et al., 2011; Archer et al., 2014; Gao et al., 2016; Linderman et al., 2017; Zhao and Park, 2017; Pandarinath et al., 2018; Duncker and Sahani, 2018; Glaser et al., 2020; Bong et al., 2020; Vyas et al., 2020; Zoltowski et al., 2020; Hu et al., 2024). A fundamental insight driving this approach is that high-dimensional neural population activity is often confined to low-dimensional manifolds (Vyas et al., 2020; Duncker and Sahani, 2021). Latent dynamical systems leverage this insight by modeling high-dimensional neural data with low-dimensional latent states, revealing underlying dynamical computations and how they result in observed behavior. Linear dynamical systems (LDS) are a common choice in this family, owing to their computational simplicity and ability to effectively approximate neural dynamics (Paninski et al., 2010; Macke et al., 2011; Archer et al., 2014; Gao et al., 2016; Soldado-Magraner et al., 2024; Jha et al., 2024).

These models, however, have a key limitation: they assume a stable mapping from the underlying dynamics to the observed neural population activity throughout a task. As a result, they may fail to capture non-stationary processes in the brain that can lead to changing neuronal representations. A notable example of such a phenomenon is representational drift, which has been observed in various brain regions, ranging from the hippocampus (Ziv et al., 2013; Rubin et al., 2015) and posterior parietal cortex (Driscoll et al., 2017) to sensory regions such as V1 (Marks and Goard, 2021). Representational drift involves a gradual change in neural representations, where neuronal correlations with sensory and behavioral variables change across potentially different timescales, even when the observed behavior stays constant (Rule et al., 2019, 2020; Driscoll et al., 2022). Mounting evidence has also shown that this drift is not simply a result of experimental confounds such as recorded neuron turnover (Driscoll et al., 2022), suggesting the possibility that the activities of individual neurons change systematically while the underlying latent dynamics stay constant.

Traditionally, representational drift has been characterized as a long-term process, with studies focusing on changes that unfold across multiple sessions spanning days or weeks. Recent evidence, however, suggests that representational drift may occur on shorter timescales than previously reported, even within individual recording sessions lasting minutes to hours (Clarke et al., 2026). With a few notable exceptions (Carmena et al., 2005; Dahmen et al., 2022), shorter timescale changes in correlated patterns of neural activity have received limited attention in neuroscience, partly due to a lack of interpretable computational tools for detecting and quantifying subtle shifts in population-level structure at finer temporal resolutions.

Motivated by this, we introduce a new model class called the Stiefel Manifold Dynamical System (SMDS), which generalizes the LDS by allowing the mapping from latent states to observations to change over time. In the SMDS, emission matrices are constrained to lie on the Stiefel manifold—the space of all orthonormal matrices—and evolve smoothly along this manifold over trials. In contrast, the dynamics parameters are held fixed across trials. Consequently, SMDS models neural activity during a task as arising from a latent space with stable underlying dynamics, while allowing the mapping from this space to the observed activity to drift over trials. The orthonormal parameterization of the latents ensures this phenomenon is identifiable and allows us to quantify drift from changes in the emission matrices. To estimate the model parameters, we developed an variational expectation-maximization algorithm with extended Kalman smoothing.

We demonstrate the effectiveness of SMDS on both simulated and real neural datasets. In simulations, SMDS models outperformed LDS, which failed to recover the true dynamics and yielded lower held-out log-likelihood than SMDS in non-stationary settings. On neural recordings from macaques and rodents, SMDS again outperformed LDS on held-out data log-likelihood, crucially requiring fewer latent dimensions to capture the activity. Using the learned emission matrices from SMDS, we computed the Grassmann distance between them across trials and found a gradual drift in both datasets. SMDS also allowed us to decompose drift along individual subspace dimensions. Peak drifts ranged 13° – 50° over 24 minutes in macaque primary motor cortex during a center-out reaching task (Clarke et al., 2026), and 37° – 67° over 30 minutes in rodent anterior lateral motor cortex during a directional licking task (Chen et al., 2017). Notably, SMDS revealed less drift in dimensions encoding higher levels of neural and behavioral variance, suggesting that task-relevant information stays relatively more stable. Overall, we show that SMDS provides a powerful framework for tracking representational drift.^1^

## 2 Background

To develop our model for capturing representational drift, we require tools from both dynamical systems and differential geometry. We first introduce linear dynamical systems (LDS), a class of models for describing latent temporal structure in time series data. We then discuss identifiability issues in LDS and show how orthogonal parameterizations address them, motivating the use of Grassmann and Stiefel manifolds as the geometric framework for modeling how the mapping from latent dynamics to observations changes over time. Finally, we briefly review the extended Kalman filter (EKF) and extended Kalman smoother (EKS) in Appendix A, which enable approximate inference in nonlinear dynamical systems.

### 2.1 Linear Dynamical Systems

A standard linear dynamical system (LDS) describes observed data $y_{t}^{\left(k\right)}\in {\mathbb{R}}^{N}$ at timestep t of trial k as arising from a low-dimensional latent state $x_{t}^{\left(k\right)}\in {\mathbb{R}}^{D}$. It consists of three main components: an initial state distribution, latent state dynamics, and an emission model.

The latent state, $x_{t}^{\left(k\right)}$, evolves over time according to a discrete-time linear Gaussian dynamics: $x_{t+1}^{\left(k\right)}=Ax_{t}^{\left(k\right)}+b+q_{t}^{\left(k\right)}$, where $A\in {\mathbb{R}}^{D\times D}$ is the state dynamics matrix, $b\in {\mathbb{R}}^{D}$ is the state bias vector, and $q_{t}^{\left(k\right)}\sim N(0,Q)$ represents dynamics noise. We sample the initial state $x_{1}^{\left(k\right)}$ at t=1 from an initial distribution $N\left(\mu_{x_{1}},\Sigma_{x_{1}}\right)$. Finally, a linear mapping from the latent state generates the observations, $y_{t}^{\left(k\right)}=Cx_{t}^{\left(k\right)}+r_{t}^{\left(k\right)}$, where $C\in {\mathbb{R}}^{N\times D}$ is the emission matrix, and $r_{t}^{\left(k\right)}\sim N(0,R)$ represents observation noise.

### 2.2 Identifiability in LDS

A key feature of LDS models is that there exists an equivalence class of parameters that all give rise to the same marginal distribution of observations. Specifically, for any invertible matrix $T\in {\mathbb{R}}^{N\times N}$, the transformed parameters ${\tilde{\Sigma }}_{x_{1}}=T\Sigma_{x_{1}}T^{⊤}$, $\tilde{A}=\mathrm{TA}T^{-1}$, $\tilde{C}=CT^{-1}$, $\tilde{Q}=\mathrm{TQ}T^{⊤}$ result in the same distribution of observations. Under this transformation, the latent states become ${\tilde{x}}_{t}^{\left(k\right)}=Tx_{t}^{\left(k\right)}$.

We can reduce non-identifiability by constraining C to be orthonormal, $C^{⊤}C=I$. Under this constraint, the only remaining transformations that preserve orthonormality are rotations within the latent space. Thus, this orthonormal parameterization identifies a unique subspace spanned by the emission matrix. This motivates the use of the Grassmann and Stiefel manifolds, which provide the natural geometric framework for working with such orthonormal matrices and their associated subspaces.

### 2.3 Grassmann and Stiefel Manifolds

The Grassmann manifold, denoted Grassmann (N,D), is the set of all D-dimensional subspaces of ${\mathbb{R}}^{N}$ (Bendokat et al., 2024). Each point on the manifold is represented by a rank-D orthogonal projection matrix:

(2.1) $$\mathrm{Grassmann}(N,D):=\left\{P\in {\mathbb{R}}^{N\times N}|P^{⊤}=P,\;P^{2}=P,\;\mathrm{rank}\;P=D\right\}.$$

Each P∈Grassmann(N,D) corresponds to a unique D-dimensional subspace of ${\mathbb{R}}^{N}$.

A given subspace can be represented by multiple different orthonormal bases—any rotation or reflection of the basis vectors spans the same subspace. The Stiefel manifold captures this collection of orthonormal basis (ONB) representations:

(2.2) $$\mathrm{Stiefel}(N,D):=\left\{C\in {\mathbb{R}}^{N\times D}|C^{⊤}C=I_{D}\right\}.$$

In our work, we parametrize the emission matrices $C^{\left(k\right)}$ as elements of the Stiefel manifold, which allows us to track their evolution over trials while maintaining orthonormality. We then use the Grassmann distance to quantify drift by measuring changes in the underlying subspaces, which is invariant to rotations within those subspaces.

#### Displacements on the Stiefel manifold.

To model how emission matrices evolve over trials while remaining orthonormal, we parametrize displacements on the Stiefel manifold using skew-symmetric matrices. At trial k, we construct a skew-symmetric matrix $B\in {\mathbb{R}}^{N\times N}$ as: $B=\left[\begin{array}{cc} W-W^{⊤} & V\\ -V^{⊤} & 0 \end{array}\right]$ where $W\in {\mathbb{R}}^{D\times D}$ is a strictly upper-triangular matrix and $V\in {\mathbb{R}}^{D\times (N-D)}$ is arbitrary. The matrix B has two geometric roles: V governs motion orthogonal to the current subspace (inducing drift on the Grassmann manifold), while W governs rotations within the subspace (which do not change the underlying subspace).

We then map B to an orthogonal matrix using the Cayley transform, f(⋅):

(2.3) $$f_{\text{Cay}}(B)=\left(I_{N}-B\right){\left(I_{N}+B\right)}^{-1}.$$

Note that we could alternatively map the skew-symmetric matrix B to an orthogonal matrix using the matrix exponential, but this is generally more costly and leads to a less well-behaved optimization problem (Wen and Yin, 2013).

#### Grassmann distance.

The Grassmann distance quantifies how far two subspaces are from each other. Given ONB matrices $C^{\left(1\right)}$, $C^{\left(2\right)}\in \text{Stiefel}(N,D)$ and principal angles $0\leq \theta_{1}\leq \theta_{2}\ldots \theta_{D}\leq \frac{\pi }{2}$ between their column spaces (Björck and Golub, 1973), the Grassmann distance is:

(2.4) $$d_{G}\left(C_{1},C_{2}\right)={\left(\sum_{d=1}^{D}\theta_{d}^{2}\right)}^{1/2}\in \left[0,\frac{\pi \sqrt{D}}{2}\right].$$

This corresponds to the geodesic distance—the shortest path between the two subspaces on the Grassmann manifold. We also define the normalized Grassmann distance as: $d_{G,\text{norm}}\left(C_{1},C_{2}\right)=d_{G}\left(C_{1},C_{2}\right)/\left(\frac{\pi \sqrt{D}}{2}\right)\in [0,1]$.

## 3 Stiefel Manifold Dynamical System

Here we introduce the Stiefel Manifold Dynamical System (SMDS). We first define the generative model and then describe our inference procedure to infer latent trajectories of the orthonormal emission matrices on the Stiefel manifold. Finally, we describe a model selection strategy based on estimated marginal log-likelihood in Appendix C.

### 3.1 Model Definition

**Figure 1:**
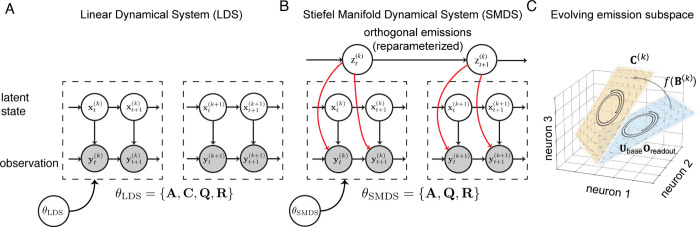
Stiefel Manifold Dynamical System. **(A-B)**: Graphical models of LDS and SMDS. At time t in trial k, $y_{t}^{\left(k\right)}\in {\mathbb{R}}^{N}$ denotes the observed neural activity, and $x_{t}^{\left(k\right)}\in {\mathbb{R}}^{D}$ denotes the underlying low-dimensional latent state. SMDS, unlike a standard LDS, assumes that the emission subspace evolves smoothly over trials, parameterized by $z^{\left(k\right)}$. **(C)**: Illustration of SMDS emission subspace evolution. $U_{\text{base}}O_{\text{readout}}$ is the fixed initial D−dimensional subspace in ${\mathbb{R}}^{N}$. $z^{\left(k\right)}$ captures the rotation at trial k relative to this, resulting in the rotated emission subspace $C^{\left(k\right)}=U_{\text{base}}f\left(B^{\left(k\right)}\right)O_{\text{readout}}$.

The Stiefel Manifold Dynamical System, as shown in Fig. 1, extends the LDS by allowing the emission matrices to vary across trials. We parameterize the evolution of the emission matrices through a latent “displacement” variable $z^{\left(k\right)}\in {\mathbb{R}}^{\frac{D(2N-D-1)}{2}}$, where k indexes the trial. We define the displacement as the concatenation of $w^{\left(k\right)}\in {\mathbb{R}}^{\frac{D(D-1)}{2}}$ and the vectorization of $V^{\left(k\right)}\in {\mathbb{R}}^{D\times (N-D)}$:

(3.1) $$z^{\left(k\right)}=\left[\begin{array}{c} w^{\left(k\right)}\\ \mathrm{vec}\left(V^{\left(k\right)}\right) \end{array}\right]$$

where $w^{\left(k\right)}$, a vector consisting of the strictly upper-triangular entries of $W^{\left(k\right)}$, governs rotations within the latent subspace, and $V^{\left(k\right)}$ governs rotations that change the subspace itself. The displacement variables evolve across trials according to a random walk,

(3.2) $$z^{\left(1\right)}\sim N\left(m_{z},S_{z}\right)$$

(3.3) $$z^{\left(k+1\right)}=z^{\left(k\right)}+e^{\left(k\right)},\;e^{\left(k\right)}\sim N\left(0,\mathrm{diag}\left(\tau_{z}^{2}\right)\right)$$

where $\tau_{z}^{2}\in {\mathbb{R}}_{>0}^{\frac{D(2N-D-1)}{2}}$ controls the rate of drift and is learned separately for each dimension so that each axis of the subspace can rotate at a different rate. The displacement variable defines a skew-symmetric matrix

(3.4) $$B^{\left(k\right)}=\left[\begin{array}{cc} W^{\left(k\right)}-{\left(W^{\left(k\right)}\right)}^{⊤} & V^{\left(k\right)}\\ -V^{{\left(k\right)}^{⊤}} & 0 \end{array}\right]$$

where $W^{\left(k\right)}=\mathrm{triu}\left(w^{\left(k\right)}\right)\in {\mathbb{R}}^{D\times D}$ is a strictly upper triangular matrix.

The emission matrix $C^{\left(k\right)}$ for trial k is then defined as:

(3.5) $$C^{\left(k\right)}=h\left(z^{\left(k\right)}\right)=U_{\text{base}}f\left(B^{\left(k\right)}\right)O_{\text{readout}}$$

where $O_{\text{readout}}=\left[\begin{array}{c} I_{D}\\ 0 \end{array}\right]\in {\mathbb{R}}^{N\times D}$, and $U_{\text{base}}$ is a fixed set of coordinate axes spanning the ambient space ${\mathbb{R}}^{N}$, in which the subspace lives and rotates. In practice, $U_{\text{base}}$ is initialized using the principal components of the training data. We choose f(⋅) to be the Cayley transform, as described in Sec. 2.3. Thus, function $h:{\mathbb{R}}^{\frac{D(2N-D-1)}{2}}\to \text{Stiefel}(N,D)$ maps the displacement variable $z^{\left(k\right)}$ to a point $C^{\left(k\right)}\in \mathrm{Stiefel}(N,D)$, with $f\left(B^{\left(k\right)}\right)$ defining the rotation matrix that transforms $U_{\text{base}}$ at each trial k, and $O_{\text{readout}}$ is a fixed matrix for reading out the first D columns (Fig. 1C).

The SMDS emission model is thus $y_{t}^{\left(k\right)}=C^{\left(k\right)}x_{t}^{\left(k\right)}+r_{t}^{\left(k\right)},r_{t}^{\left(k\right)}\sim N(0,R)$, where the emission matrix varies over trials. The dynamics parameters {A,Q} are shared over trials, as in a standard LDS (Sec. 2.1). To complete the model specification, we place an Inverse-gamma prior on each entry of $\tau_{z}^{2}$, where the prior parameters are chosen via cross-validation (Appendix J).

### 3.2 Latent State and Parameter Inference

SMDS has two sets of latent variables: the within-trial latent states ${\left\{x_{1:T^{\left(k\right)}}^{\left(k\right)}\right\}}_{k=1}^{K}$ and the across-trial displacements ${\left\{z^{\left(k\right)}\right\}}_{k=1}^{K}$, which parameterize the emission matrices. Computing the exact joint posterior over both sets of latent variables is intractable. Thus, we use approximate coordinate ascent variational inference under a structured mean-field factorization (Ghahramani and Hinton, 2000; Blei et al., 2017). We assume a factorized approximate posterior,

(3.6) $$q\left({\left\{x_{1:T^{\left(k\right)}}^{\left(k\right)}\right\}}_{k=1}^{K},{\left\{z^{\left(k\right)}\right\}}_{k=1}^{K}\right)=q\left({\left\{z^{\left(k\right)}\right\}}_{k=1}^{K}\right)\prod_{k=1}^{K}q\left(x_{1:T^{\left(k\right)}}^{\left(k\right)}\right),$$

and alternate between updating each factor while holding the other fixed, interleaved with M-step updates of the model parameters $\left\{\mu_{x_{1}},\Sigma_{x_{1}},A,Q,R\right\}$.

#### Updating $q\left({\left\{x_{1:T^{\left(k\right)}}^{\left(k\right)}\right\}}_{k=1}^{K}\right)$: Kalman smoothing.

Given the current estimate of the displacements, we fix the emission matrices at ${\hat{C}}^{\left(k\right)}=h\left({\hat{z}}^{\left(k\right)}\right)$, where ${\hat{z}}^{\left(k\right)}=E_{q\left({\left\{z^{\left(k\right)}\right\}}_{k=1}^{K}\right)}\left[z^{\left(k\right)}\right]$ is the posterior mean. While it is suboptimal to condition on only a point estimate of the emission matrix rather than the propagating uncertainty from the variational factor, $q\left({\left\{z^{\left(k\right)}\right\}}_{k=1}^{K}\right)$, this approach is much more straightforward. Given a point estimate, the update for the within-trial latent states reduces to a standard LDS, and we update the factor $q\left(x_{1:T^{\left(k\right)}}^{\left(k\right)}\right)$ using exact Kalman smoothing for each trial k.

#### Updating $q\left({\left\{z^{\left(k\right)}\right\}}_{k=1}^{K}\right)$: Extended Kalman smoothing.

Given the current $q\left(x_{1:T^{\left(k\right)}}^{\left(k\right)}\right)$ for k=1,…,K from the previous step, we update the approximate posterior over the displacements. With the structured mean field approximation, the optimal update is,

(3.7) $$\log q^{*}\left({\left\{z^{\left(k\right)}\right\}}_{k=1}^{K}\right)=E_{q\left({\left\{x_{1:T^{\left(k\right)}}^{\left(k\right)}\right\}}_{k=1}^{K}\right)}\left[\log p\left({\left\{y_{1:T^{\left(k\right)}}^{\left(k\right)}\right\}}_{k=1}^{K},{\left\{x_{1:T^{\left(k\right)}}^{\left(k\right)}\right\}}_{k=1}^{K},{\left\{z^{\left(k\right)}\right\}}_{k=1}^{K}|\theta \right)\right]+\text{const}.$$

As derived in Appendix B, the expected log joint probability on the right-hand side is equivalent to a state space model with linear Gaussian dynamics (from the prior on $\left.{\left\{z^{\left(k\right)}\right\}}_{k=1}^{K}\right)$ and *nonlinear* Gaussian emissions,

(3.8) $${\hat{y}}_{z}^{\left(k\right)}\sim N\left(\mathrm{vec}\left(h\left(z^{\left(k\right)}\right)\right),{\hat{R}}_{z}^{\left(k\right)}\right),$$

where ${\hat{R}}_{z}^{\left(k\right)}={\left(R^{-1}⊗\sum_{t=1}^{T^{\left(k\right)}}E_{q}\left[x_{t}^{\left(k\right)}x_{t}^{(k)⊤}\right]\right)}^{-1}$, ${\hat{y}}_{z}^{\left(k\right)}={\hat{R}}_{z}^{\left(k\right)}\mathrm{vec}\left(R^{-1}\sum_{t=1}^{T^{\left(k\right)}}E_{q}\left[y_{t}^{\left(k\right)}x_{t}^{(k)⊤}\right]\right)$, and, ⊗ denotes the Kronecker product. Again, the exact coordinate ascent update is challenging to compute. Instead, noting that the emission function vec(h(⋅)) is nonlinear, we propose to use an extended Kalman smoother (EKS) to compute an approximate Gaussian posterior $q\left({\left\{z^{\left(k\right)}\right\}}_{k=1}^{K}\right)$, analogous to previous work (Zoltowski et al., 2020).

#### M-step.

Given the approximate posteriors $q\left({\left\{x_{1:T^{\left(k\right)}}^{\left(k\right)}\right\}}_{k=1}^{K}\right)$ and $q\left({\left\{z^{\left(k\right)}\right\}}_{k=1}^{K}\right)$, we update the model parameters. The dynamics and observation noise parameters $\left\{\mu_{x_{1}},{\text{Σ}}_{x_{1}},A,Q,R\right\}$ are updated in closed form, as in a standard LDS. The displacement prior parameters $\left\{m_{z},S_{z}\right\}$ and drift rates $\tau_{z}^{2}$ are updated using the posterior statistics from $q\left({\left\{z^{\left(k\right)}\right\}}_{k=1}^{K}\right)$. We find that the quality of the first-order approximation used in the EKS depends on the magnitude of $\tau_{z}^{2}$ (see details in Appendix D). As such, we clip $\tau_{z}^{2}$ after each M step, where the clipping threshold is also a tunable hyperparameter (Appendix J).

## 4 Results

We first compare SMDS to LDS on synthetically generated non-stationary data. Next, we apply SMDS, LDS, and conditionally linear dynamical systems (CLDS) (Geadah et al., 2025) to analyze real macaque neural recordings during a center-out reaching task and rodent two-photon calcium imaging data during a whisker-based directional licking task. CLDS uses Gaussian process priors to allow model parameters to vary smoothly with observed covariates such as reaching directions. We adapt CLDS to model representational drift by setting the covariate to the trial block index and allowing only the emission matrix to vary across trials, while keeping the dynamics parameters fixed (see Appendix F for details and Appendix J for hyperparameters).

### 4.1 Application to simulated data

**Figure 2:**
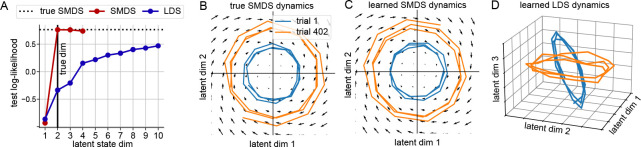
Simulated data experiment 1 **(A)**: Held-out log-likelihood on data generated from an SMDS with D=2, N=10. SMDS outperforms standard LDS and recovers the true state dimension. **(B–D)**: SMDS fitted to this data with D=2 recovers ground truth dynamics, while LDS requires higher-dimensional states.

We begin with a toy example to visually demonstrate the advantages of SMDS over a standard LDS. We simulated data from SMDS with latent dimension D=2 and observation dimension N=10. The simulated dataset consisted of a total of 750 trials, with 30 timesteps per trial, where the emission matrices smoothly rotated over trials (see details in Appendix G.1). We fitted both LDS and SMDS across a range of state dimensions, D∈[1,10], and evaluated model performance based on held-out data log-likelihood (Fig. 2A).

We found that the test log-likelihood of SMDS saturated at the ground truth latent dimensionality, and that SMDS achieved higher test log-likelihood than LDS. The test log-likelihood of LDS continued to increase beyond the true latent dimensionality, failing to identify the correct dimensionality. We also visualized the true and learned smoothed latent trajectories for two example trials (Fig. 2B). While SMDS recovered the true two-dimensional dynamics up to a rotation (Fig. 2C), the LDS with D=3 learned trial-specific shifted dynamics (Fig. 2D) to compensate for drift in the observation space. Although the test log-likelihood for LDS kept increasing, we chose D=3 for visualization purposes. We show in Appendix G.2 that SMDS also recovers ground-truth dynamics and drift in higher-dimensional settings (D=8,N=24), including per-dimension drift rates.

### 4.2 Modeling macaque neural data during center-out reaching task

**Figure 3:**
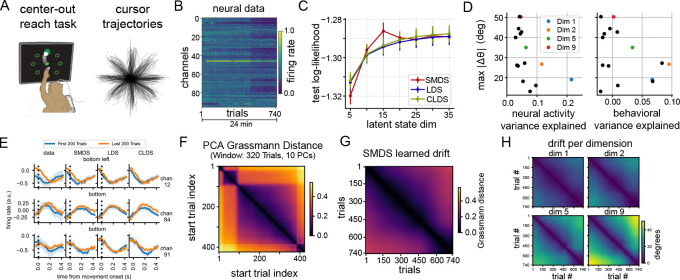
SMDS drift analysis on neural recordings from a macaque performing a center-out reaching task. **(A)** Task schematic and example center-out reach trajectories. **(B)** Raster plot of neural activity from a single session. The dataset includes recordings from 96 channels, with a total of 744 trials. **(C)** Held-out log-likelihood comparison. SMDS outperforms LDS and CLDS using fewer latent dimensions. **(D)** Peak drift magnitude during the session vs. explained variance per dimension: neural (left) and behavioral (right). Axes that account for more neural variance drift less, whereas less informative axes drift more **(E)** PSTHs from early and late trials for a subset of channels under the “bottom” and “bottom left” reach conditions, aligned to movement onset, showing evidence of representational drift (left to right: data, SMDS, LDS, and CLDS predictions). **(F)** Normalized Grassmann distance matrix computed using the top 10 principal components over a sliding window of 320 trials. **(G)** Overall drift inferred by SMDS, computed using Grassmann distance between pairs of learned emission matrices. **(H)** Estimated drift per dimension, computed in degrees.

We applied SMDS to neural recordings from Clarke et al. (2026),^2^ where a macaque performed a center-out reaching task (Fig. 3A) The dataset comprised 96-channel Utah array multiunit activity from M1 spanning 750 trials from a post-lesion session (Fig. 3B). Trials were grouped in blocks of eight, each containing all conditions in random order. We binned neural activity in 25 ms bins and performed our analysis on activity from −50 ms to 450 ms relative to movement onset.

We found evidence of representational drift through both visual investigation and principal component analysis of the raw data. Fig. 3B & E (left) show average firing rates over trials and peri-stimulus time histograms (PSTHs), respectively, for three example channels. Both firing rates and PSTHs change between early and late trials. Additionally, the normalized Grassmann distance between subspaces spanned by the top 10 principal components (PCs), computed using sliding windows of 320 trials, revealed subspace rotation indicative of drift (Fig. 3F).

We fitted SMDS, LDS, and CLDS using 5–45 latent dimensions across five train-test splits, holding out 12 random blocks per split. We allowed the emission matrices to evolve across blocks in SMDS and CLDS (see Appendix H for all hyperparameter selection details). SMDS outperformed both LDS and CLDS in terms of marginal log-likelihood on held-out blocks, saturating at a smaller latent dimensionality (Fig. 3C), highlighting its ability to model non-stationary data effectively. Predicted PSTHs from SMDS also closely matched real data, as shown in Fig. 3E.

To characterize the drift learned by SMDS, we computed the normalized Grassmann distance between emission subspaces across blocks, revealing a gradual drift throughout the session (Fig. 3G). Decomposing the drift along individual latent dimensions, we found that dimensions explaining more variance in the neural data (Fig. 3D left) and those that are behaviorally significant—in terms of predicting cursor velocity—exhibited less drift (Fig. 3D right). Conversely, less informative dimensions showed more drift. Visualization of pairwise drift between blocks for selected latent dimensions (Fig. 3H) revealed varying patterns. Some dimensions (e.g., 9) rotated up to 50° and showed structured drift patterns similar to the PC subspace drift (Fig. 3D & F), while others (e.g., 1) exhibited more modest drift around 19°.

Thus, these findings reveal gradual representational drift in macaque M1 during the reach task. However, this drift is not uniform across all latent dimensions. Instead, it appears to preferentially affect less neurally and behaviorally significant dimensions, potentially to maintain stable representations of task-relevant information over time.

### 4.3 Modeling rodent neural data during a directional licking task

**Figure 4:**
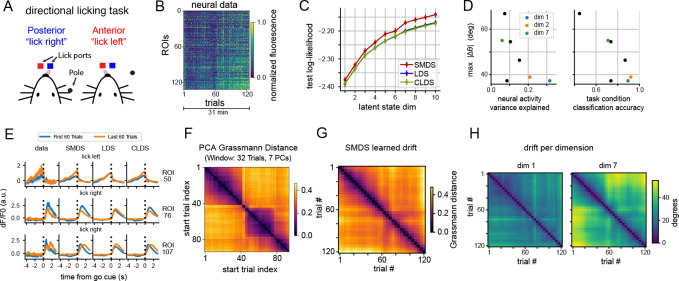
SMDS drift analysis on a rodent neural dataset during a directional licking task. **(A)** Task schematic. The rodent licks either the left or right port based on the pole’s location. **(B)** Two-photon calcium imaging fluorescence from a single session. The session includes recordings from 132 ROIs over approximately 31 minutes, consisting of 155 trials. We analyzed 126 correct trials with no early licks. **(C)** Held-out log-likelihood comparison showing that SMDS consistently outperforms standard LDS. **(D)** Peak drift magnitude vs explained neural variance per dimension (left), and trial condition classification accuracy (right). Axes that account for more neural and behavioral variance drift less. **(E)** PSTHs from early and late trials for a subset of ROIs, aligned to the “go” cue (left to right: data, SMDS, LDS, and CLDS predictions) **(F)** Normalized Grassmann distance matrix computed using the top 7 principal components over a sliding window of 32 trials. **(G)** Overall drift inferred by SMDS, computed using Grassmann distance between pairs of learned emission matrices. **(H)** Estimated drift per dimension, computed in degrees.

We next applied SMDS to a two-photon calcium imaging dataset from Chen et al. (2017), where a rodent performed a whisker-based object localization task with a delayed, directional licking response (Fig. 4A). The dataset included 126 correct trials from a total of 132 regions of interest (ROIs) in the anterior lateral cortex (ALM) and medial motor cortex (MM), imaged at 14Hz. We analyzed 7 seconds of data around the go cue per trial (98 time bins/trial). As with the macaque dataset, we saw evidence of changes in neural representations within the session through two analyses of the raw data: (1) visual inspection of trial-averaged responses (Fig. 4B) and PSTHs during early and late trials (Fig. 4E left), and (2) pairwise normalized Grassmann distance between the subspaces spanned by the top 7 PCs, using sliding windows of 32 trials (Fig. 4F).

We then fitted SMDS, LDS, and CLDS using 1–10 latent dimensions across three train-test splits (see Appendix I for hyperparameter details). For SMDS and CLDS, we grouped trials into blocks of four, allowing emissions to evolve over blocks. Once again, SMDS outperformed both LDS and CLDS in terms of marginal log-likelihood on held-out blocks and required fewer latent dimensions (Fig. 4C). We also visualized PSTHs predicted by SMDS (Fig. 4E) and found that they closely match the data.

We then analyzed the learned subspace changes along individual latent dimensions. Similar to the macaque dataset, dimensions that explained more neural variance and better classified trial conditions exhibited smaller peak drift (Fig. 4D), reinforcing our hypothesis that representations of task-relevant information tend to remain more stable. Visualization of pairwise normalized Grassmann distances between the learned emission subspaces over blocks revealed a gradual drift over the session (Fig. 4G). When visualizing drift for selected latent dimensions, some dimensions exhibited high and structured drift—for example, dimension 7 rotated by over 55° and showed structured drift patterns similar to the PC subspace drift (Fig. 4F)—while others, such as dimensions 1 and 2, showed slower drift with a peak around 37° (Fig. 4H). These findings emphasize that different latent dimensions drift at varying rates and patterns, where neurally and behaviorally significant dimensions tend to be more stable.

## 5 Related Work

### Subspace Tracking

Subspace tracking, the problem of dynamically estimating the subspace of streaming data, has a long history, from Oja’s algorithm (Oja, 1982) to modern methods handling high-dimensionality, limited memory, and data corruption (Yang, 1995; Balzano et al., 2018; Narayanamurthy and Vaswani, 2018a,b; Bienstock et al., 2022). Recent approaches model subspace evolution on manifolds: Sasfi et al. (2024) used Grassmannian gradient descent for subspace tracking in LDS, Blocker et al. (2023) estimated piecewise geodesic subspace trajectories, and Srivastava and Klassen (2004) and Rentmeesters et al. (2010) used particle filtering on the Grassmann manifold. Related Stiefel manifold methods employ matrix Langevin noise models with particle filtering or iterative Bayesian updates (Tompkins and Wolfe, 2007; Bordin and Bruno, 2020; Chikuse, 2006). SMDS is related to these approaches in that it allows the emission matrices to evolve on a Stiefel manifold, but differs in that it performs inference using an extended Kalman smoother.

### Analysis of Nonstationary Neural Data

Prior work addresses neural nonstationarity through alignment methods that reveal stable structure across sessions (Gallego et al., 2020), switching models that capture discrete transitions between dynamical regimes (Ghahramani and Hinton, 2000; Linderman et al., 2017; Glaser et al., 2020; Costacurta et al., 2022; Lee et al., 2023), sparse decompositions of nonstationary dynamics (Mudrik et al., 2024), and condition-dependent subspace models where parameters vary smoothly with task variables (Nejatbakhsh et al., 2023; Geadah et al., 2025). Our focus is to model changes in the observed neural representation space, while the underlying dynamics stay constant.

## 6 Discussion

We introduced the Stiefel Manifold Dynamical System (SMDS), a novel class of probabilistic state space models that learns neural dynamics while accounting for representational drift. SMDS constrains its emission matrices to be orthonormal and allows them to evolve smoothly over trials on the Stiefel manifold. On simulated data, SMDS recovered true latent dynamics and outperformed LDS, which failed to account for non-stationarity. On macaque and rodent neural recordings, SMDS revealed gradual drift occurring over a single experimental session. Our approach also revealed that dimensions encoding higher neural and behavioral variance drifted less relative to others, thus maintaining stable task-relevant representations. These findings underscore SMDS’s potential for understanding neural dynamics and their evolution over time and open new avenues for investigating sources and causes of representational drift.

Several directions remain for future work. While SMDS assumes smooth drift over trials, it may struggle to capture abrupt or event-driven drift in neural activity. Future work could exploit discrete switching transitions in drift rates to address this. Next, SMDS could be extended to relax the shared dynamics assumption, thus allowing the model to learn changes in the underlying neural computations over time, along with drift in the observation space. Finally, SMDS models the evolution of neural subspaces independently of behavior. Jointly modeling neural and behavioral data may enable SMDS to learn a time-varying mapping between the two. Despite these limitations, SMDS provides a principled framework for quantifying representational drift and understanding neural dynamics in non-stationary settings.

## Supplementary Material

Supplement 1

## Footnotes

1 Code for the SMDS model and experiments is available at https://github.com/lindermanlab/smds.

2 Please refer to Clarke et al. (2026) for further details on the dataset and instructions on data access. The dataset ID used in this work is U201202_01.
