# Supplementary material for: Stiefel Manifold Dynamical Systems for Tracking Representational Drift
Source: bioRxiv. 2026 Mar 10:2026.03.07.710319. Preprint. [Version 1] doi: 10.64898/2026.03.07.710319 (PMC13060931; doi:10.64898/2026.03.07.710319)
Supplement: Supplement 1 [file NIHPP2026.03.07.710319v1-supplement-1.pdf]

bioRxiv preprint doi: https://doi.org/10.64898/2026.03.07.710319; this version posted March 10, 2026. The copyright holder for this preprint (which was not certified by peer review) is the author/funder, who has granted bioRxiv a license to display the preprint in perpetuity. It is made available under aCC-BY 4.0 International license.

Appendix

Table of Contents

– **Appendix A**: Extended Kalman Filters and Smoothers

– **Appendix B**: Derivation of the Displacement Update in SMDS

– **Appendix C**: Computing Marginal Log-likelihood of Data under an SMDS

– **Appendix D**: First-order Approximation of the SMDS Emission Model

– **Appendix E**: Max $|\Delta\theta|$ (Peak Drift) Computation

– **Appendix F**: Conditionally Linear Dynamical Systems

– **Appendix G**: Simulated Data Experiments

– **Appendix H**: Modeling Macaque Neural Data

– **Appendix I**: Modeling Rodent Neural Data

– **Appendix J**: Experimental Configurations

– **Appendix K**: PCA Fails to Recover Drift Per Dimension

– **Appendix L**: Code Availability

bioRxiv preprint doi: https://doi.org/10.64898/2026.03.07.710319; this version posted March 10, 2026. The copyright holder for this preprint (which was not certified by peer review) is the author/funder, who has granted bioRxiv a license to display the preprint in perpetuity. It is made available under aCC-BY 4.0 International license.

A Extended Kalman Filters and Smoothers

While Kalman filters and smoothers are well-suited for linear systems, they are not directly applicable when either the dynamics or the emission models are nonlinear. Here we briefly describe the extended Kalman filter (EKF) and extended Kalman smoother (EKS), which are recursive algorithms for approximate inference in systems with nonlinear dynamics or nonlinear observation models (Särkkä, 2013).

The EKF linearizes the dynamics and observation functions around the current state estimate at each timestep using their first-order Taylor approximation, thus allowing a local application of the standard Kalman filter update. Specifically, given a nonlinear state transition function $\mathbf{x}_{t+1}^{(k)} = \phi(\mathbf{x}_t^{(k)}) + \mathbf{q}_t^{(k)}$ and emission model $\mathbf{y}_t^{(k)} = \psi(\mathbf{x}_t^{(k)}) + \mathbf{r}_t^{(k)}$, the EKF computes the Jacobians of ϕ and ψ at the current estimate and propagates the mean and covariance of the state forward in time. These linearizations yield an approximate Gaussian posterior over the latent state at each timestep, enabling efficient filtering in otherwise intractable nonlinear models. Analogous to Kalman smoothing, the EKS builds on the EKF by incorporating future observations to refine the latent state estimates.

bioRxiv preprint doi: https://doi.org/10.64898/2026.03.07.710319; this version posted March 10, 2026. The copyright holder for this preprint (which was not certified by peer review) is the author/funder, who has granted bioRxiv a license to display the preprint in perpetuity. It is made available under aCC-BY 4.0 International license.

B Derivation of the Displacement Update in SMDS

We derive the update for the approximate posterior over the displacements $q(\{\mathbf{z}^{(k)}\}_{k=1}^{K})$ used in Sec. 3.2. As described in Sec. 3.2, we assume a mean-field factorization

$$q(\{\mathbf{x}_{1:T^{(k)}}^{(k)}\}_{k=1}^{K}, \{\mathbf{z}^{(k)}\}_{k=1}^{K}) = q(\{\mathbf{z}^{(k)}\}_{k=1}^{K}) \prod_{k=1}^{K} q(\mathbf{x}_{1:T^{(k)}}^{(k)}) \tag{B.1}$$

and optimize the ELBO by approximate coordinate ascent, alternating between the two factors. The ELBO is

$$\begin{aligned} \mathcal{L} = {} & \mathbb{E}_{q(\{\mathbf{x}_{1:T^{(k)}}^{(k)}\}_{k=1}^{K})\, q(\{\mathbf{z}^{(k)}\}_{k=1}^{K})}[\log p(\{\mathbf{y}_{1:T^{(k)}}^{(k)}\}_{k=1}^{K}, \{\mathbf{x}_{1:T^{(k)}}^{(k)}\}_{k=1}^{K}, \{\mathbf{z}^{(k)}\}_{k=1}^{K} \mid \boldsymbol{\theta})] \\ & - \mathbb{E}_{q(\{\mathbf{x}_{1:T^{(k)}}^{(k)}\}_{k=1}^{K})}[\log q(\{\mathbf{x}_{1:T^{(k)}}^{(k)}\}_{k=1}^{K})] - \mathbb{E}_{q(\{\mathbf{z}^{(k)}\}_{k=1}^{K})}[\log q(\{\mathbf{z}^{(k)}\}_{k=1}^{K})]. \end{aligned} \tag{B.2}$$

Holding $q(\{\mathbf{x}_{1:T^{(k)}}^{(k)}\}_{k=1}^{K})$ fixed and optimizing over $q(\{\mathbf{z}^{(k)}\}_{k=1}^{K})$, the terms that depend on $q(\{\mathbf{z}^{(k)}\}_{k=1}^{K})$ are

$$\mathcal{L}_{q(\{\mathbf{z}^{(k)}\}_{k=1}^{K})} = \mathbb{E}_{q(\{\mathbf{z}^{(k)}\}_{k=1}^{K})}[f(\{\mathbf{z}^{(k)}\}_{k=1}^{K})] - \mathbb{E}_{q(\{\mathbf{z}^{(k)}\}_{k=1}^{K})}[\log q(\{\mathbf{z}^{(k)}\}_{k=1}^{K})], \tag{B.3}$$

where $f(\{\mathbf{z}^{(k)}\}_{k=1}^{K}) = \mathbb{E}_{q(\{\mathbf{x}_{1:T^{(k)}}^{(k)}\}_{k=1}^{K})}[\log p(\{\mathbf{y}_{1:T^{(k)}}^{(k)}\}_{k=1}^{K}, \{\mathbf{x}_{1:T^{(k)}}^{(k)}\}_{k=1}^{K}, \{\mathbf{z}^{(k)}\}_{k=1}^{K} \mid \boldsymbol{\theta})]$. Defining $\tilde{p}(\{\mathbf{z}^{(k)}\}_{k=1}^{K}) \propto \exp(f(\{\mathbf{z}^{(k)}\}_{k=1}^{K}))$, we can rewrite (B.3) as

$$\mathcal{L}_{q(\{\mathbf{z}^{(k)}\}_{k=1}^{K})} = -\mathrm{KL}(q(\{\mathbf{z}^{(k)}\}_{k=1}^{K}) \parallel \tilde{p}(\{\mathbf{z}^{(k)}\}_{k=1}^{K})) + \text{const}, \tag{B.4}$$

which is maximized when $q^*(\{\mathbf{z}^{(k)}\}_{k=1}^{K}) = \tilde{p}(\{\mathbf{z}^{(k)}\}_{k=1}^{K})$, i.e.,

$$\log q^*(\{\mathbf{z}^{(k)}\}_{k=1}^{K}) = \mathbb{E}_{q(\{\mathbf{x}_{1:T^{(k)}}^{(k)}\}_{k=1}^{K})}[\log p(\{\mathbf{y}_{1:T^{(k)}}^{(k)}\}_{k=1}^{K}, \{\mathbf{x}_{1:T^{(k)}}^{(k)}\}_{k=1}^{K}, \{\mathbf{z}^{(k)}\}_{k=1}^{K} \mid \boldsymbol{\theta})] + \text{const}. \tag{B.5}$$

bioRxiv preprint doi: https://doi.org/10.64898/2026.03.07.710319; this version posted March 10, 2026. The copyright holder for this preprint (which was not certified by peer review) is the author/funder, who has granted bioRxiv a license to display the preprint in perpetuity. It is made available under aCC-BY 4.0 International license.

Expanding the joint and dropping terms that do not depend on $\{\mathbf{z}^{(k)}\}_{k=1}^{K}$,

$$\log q^*(\{\mathbf{z}^{(k)}\}_{k=1}^{K}) = \sum_{k=1}^{K} \mathbb{E}_{q(\mathbf{x}^{(k)}_{1:T^{(k)}})}[\log p(\mathbf{y}^{(k)}_{1:T^{(k)}} \mid \mathbf{x}^{(k)}_{1:T^{(k)}}, \mathbf{z}^{(k)})] + \log p(\{\mathbf{z}^{(k)}\}_{k=1}^{K}) + \text{const.} \tag{B.6}$$

Below we show that (B.6) takes the form of a state-space model with a nonlinear emission function, allowing us to approximate q^* with an extended Kalman smoother. We begin by simplifying the first term.

Per-trial emission log-likelihood We begin with the emission log-likelihood for a single trial k, conditioned on the latent states and the displacement:

$$\log p(\mathbf{y}^{(k)}_{1:T^{(k)}} \mid \mathbf{x}^{(k)}_{1:T^{(k)}}, \mathbf{z}^{(k)}) = -\frac{1}{2} \sum_{t=1}^{T^{(k)}} (\mathbf{y}^{(k)}_t - h(\mathbf{z}^{(k)})\, \mathbf{x}^{(k)}_t)^\top \mathbf{R}^{-1} (\mathbf{y}^{(k)}_t - h(\mathbf{z}^{(k)})\, \mathbf{x}^{(k)}_t) + \text{const.} \tag{B.7}$$

Expanding the quadratic form and dropping terms that do not depend on $\mathbf{z}^{(k)}$,

$$\begin{aligned} &\sum_{t=1}^{T^{(k)}} (\mathbf{y}^{(k)}_t - h(\mathbf{z}^{(k)})\, \mathbf{x}^{(k)}_t)^\top \mathbf{R}^{-1} (\mathbf{y}^{(k)}_t - h(\mathbf{z}^{(k)})\, \mathbf{x}^{(k)}_t) \\ &\quad = \sum_{t=1}^{T^{(k)}} \Big[\mathbf{x}^{(k)\top}_t h(\mathbf{z}^{(k)})^\top \mathbf{R}^{-1}\, h(\mathbf{z}^{(k)})\, \mathbf{x}^{(k)}_t - 2\, \mathbf{y}^{(k)\top}_t \mathbf{R}^{-1}\, h(\mathbf{z}^{(k)})\, \mathbf{x}^{(k)}_t\Big] + \text{const.} \end{aligned} \tag{B.8}$$

Vectorized form Using the identities $\operatorname{tr}(\mathbf{X}\mathbf{A}\mathbf{X}^\top\mathbf{B}) = \operatorname{vec}(\mathbf{X})^\top(\mathbf{B} \otimes \mathbf{A})\operatorname{vec}(\mathbf{X})$ (for symmetric $\mathbf{A}$ and $\mathbf{B}$) and $\operatorname{tr}(\mathbf{A}^\top\mathbf{B}) = \operatorname{vec}(\mathbf{A})^\top\operatorname{vec}(\mathbf{B})$, we rewrite (B.8) in terms of

bioRxiv preprint doi: https://doi.org/10.64898/2026.03.07.710319; this version posted March 10, 2026. The copyright holder for this preprint (which was not certified by peer review) is the author/funder, who has granted bioRxiv a license to display the preprint in perpetuity. It is made available under aCC-BY 4.0 International license.

$\mathrm{vec}(h(\mathbf{z}^{(k)}))$:

$$= \sum_{t=1}^{T^{(k)}} \Big[\mathrm{vec}(h(\mathbf{z}^{(k)}))^\top (\mathbf{R}^{-1} \otimes \mathbf{x}_t^{(k)} \mathbf{x}_t^{(k)\top}) \,\mathrm{vec}(h(\mathbf{z}^{(k)})) - 2\,\mathrm{vec}(\mathbf{R}^{-1} \mathbf{y}_t^{(k)} \mathbf{x}_t^{(k)\top})^\top \mathrm{vec}(h(\mathbf{z}^{(k)})) \Big] + \mathrm{const}$$

$$= \mathrm{vec}(h(\mathbf{z}^{(k)}))^\top \Big(\mathbf{R}^{-1} \otimes \sum_{t=1}^{T^{(k)}} \mathbf{x}_t^{(k)} \mathbf{x}_t^{(k)\top} \Big) \mathrm{vec}(h(\mathbf{z}^{(k)})) \;-\; 2\,\mathrm{vec}\Big(\mathbf{R}^{-1} \sum_{t=1}^{T^{(k)}} \mathbf{y}_t^{(k)} \mathbf{x}_t^{(k)\top} \Big)^\top \mathrm{vec}(h(\mathbf{z}^{(k)})) + \mathrm{const}. \tag{B.9}$$

Completing the square Taking expectations under $q(\{\mathbf{x}_{1:T^{(k)}}^{(k)}\}_{k=1}^{K})$ and completing the square in $\mathrm{vec}(h(\mathbf{z}^{(k)}))$, the expected emission log-likelihood (B.7) is equivalent (up to a constant) to

$$-\frac{1}{2}(\hat{\mathbf{y}}_z^{(k)} - \mathrm{vec}(h(\mathbf{z}^{(k)})))^\top (\hat{\mathbf{R}}_z^{(k)})^{-1} (\hat{\mathbf{y}}_z^{(k)} - \mathrm{vec}(h(\mathbf{z}^{(k)}))), \tag{B.10}$$

where

$$\hat{\mathbf{R}}_z^{(k)} = \Big(\mathbf{R}^{-1} \otimes \sum_{t=1}^{T^{(k)}} \mathbb{E}_q[\mathbf{x}_t^{(k)} \mathbf{x}_t^{(k)\top}] \Big)^{-1}, \tag{B.11}$$

$$\hat{\mathbf{y}}_z^{(k)} = \hat{\mathbf{R}}_z^{(k)}\, \mathrm{vec}\Big(\mathbf{R}^{-1} \sum_{t=1}^{T^{(k)}} \mathbb{E}_q[\mathbf{y}_t^{(k)} \mathbf{x}_t^{(k)\top}] \Big). \tag{B.12}$$

Here, $\otimes$ denotes the Kronecker product.

State-space model for the displacements Substituting (B.10) into (B.6), we can interpret the right-hand side as the log joint of a state-space model with linear dynamics

bioRxiv preprint doi: https://doi.org/10.64898/2026.03.07.710319; this version posted March 10, 2026. The copyright holder for this preprint (which was not certified by peer review) is the author/funder, who has granted bioRxiv a license to display the preprint in perpetuity. It is made available under aCC-BY 4.0 International license.

(from the random walk prior $\log p(\{\mathbf{z}^{(k)}\}_{k=1}^{K})$) and a nonlinear emission function:

$$\text{Initial distribution:} \quad \mathbf{z}^{(1)} \sim \mathcal{N}(\mathbf{m}_z, \mathbf{S}_z), \tag{B.13}$$

$$\text{Linear dynamics:} \quad \mathbf{z}^{(k+1)} \sim \mathcal{N}(\mathbf{z}^{(k)}, \operatorname{diag}(\boldsymbol{\tau}_z^2)), \tag{B.14}$$

$$\text{Nonlinear emissions:} \quad \hat{\mathbf{y}}_z^{(k)} \sim \mathcal{N}(\operatorname{vec}(h(\mathbf{z}^{(k)})), \hat{\mathbf{R}}_z^{(k)}). \tag{B.15}$$

Concretely, since $\{\hat{\mathbf{y}}_z^{(k)}\}_{k=1}^{K}$ are fixed quantities (computed from the data and the current $q(\{\mathbf{x}_{1:T^{(k)}}^{(k)}\}_{k=1}^{K})$), we have

$$\log q^*(\{\mathbf{z}^{(k)}\}_{k=1}^{K}) = \log p(\{\mathbf{z}^{(k)}\}_{k=1}^{K}, \mid \{\hat{\mathbf{y}}_z^{(k)}\}_{k=1}^{K}) + \text{const}, \tag{B.16}$$

We approximate q^* using an extended Kalman smoother, yielding an approximate Gaussian posterior $q(\{\mathbf{z}^{(k)}\}_{k=1}^{K})$. The posterior mean $\hat{\mathbf{z}}^{(k)}$ gives the estimated emission matrix $\hat{\mathbf{C}}^{(k)} = h(\hat{\mathbf{z}}^{(k)})$.

bioRxiv preprint doi: https://doi.org/10.64898/2026.03.07.710319; this version posted March 10, 2026. The copyright holder for this preprint (which was not certified by peer review) is the author/funder, who has granted bioRxiv a license to display the preprint in perpetuity. It is made available under aCC-BY 4.0 International license.

C Computing Marginal Log-likelihood of Data under an SMDS

To evaluate and compare the performance of SMDS with a standard LDS, we approximate the marginal log-likelihood of held-out data given the training data to test the model's ability to generalize to unseen data. Approximating the marginal log-likelihood for an SMDS model requires marginalizing over both latent states and displacements. Naïvely, this would require memory of $\mathcal{O}(N^2T^2)$ where N is the data dimensionality and T is the length of a trial (Appendix C.1). Thus, for computational and memory efficiency, we formulated an equivalent representation of SMDS in an augmented latent space that jointly includes the latent states and displacements (Appendix C.2). This eliminates the need to store statistics for each time point in memory and allows us to use an EKF in the augmented space and obtain marginal log-likelihood estimates efficiently.

C.1 Block-wise Approach

Let $\mathcal{Y} \in \mathbb{R}^{K \times T \times N}$ be the tensor denoting the data. Here, we describe the naïve approach to approximate the marginal log-likelihood. Omitting the dependence on the learned

bioRxiv preprint doi: https://doi.org/10.64898/2026.03.07.710319; this version posted March 10, 2026. The copyright holder for this preprint (which was not certified by peer review) is the author/funder, who has granted bioRxiv a license to display the preprint in perpetuity. It is made available under aCC-BY 4.0 International license.

parameters for simplicity, we can approximate it as follows:

$$
\begin{aligned}
&\log p(\mathcal{Y}) \\
&= \sum_{k=1}^{K} \log p\left(\mathbf{Y}^{(k)} \mid \mathbf{Y}^{(1:k-1)}\right) \\
&= \sum_{k=1}^{K} \log \int p\left(\mathbf{Y}^{(k)} \mid \mathbf{z}^{(k)}\right) p\left(\mathbf{z}^{(k)} \mid \mathbf{Y}^{(1:k-1)}\right) d\mathbf{z}^{(k)} \\
&= \sum_{k=1}^{K} \log \int \left[\prod_{t=1}^{T^{(k)}} p\left(\mathbf{y}_t^{(k)} \mid \mathbf{y}_{1:t-1}^{(k)}, \mathbf{z}^{(k)}\right)\right] p\left(\mathbf{z}^{(k)} \mid \mathbf{Y}^{(1:k-1)}\right) d\mathbf{z}^{(k)} \\
&= \sum_{k=1}^{K} \log \int \left[\prod_{t=1}^{T^{(k)}} \int p\left(\mathbf{y}_t^{(k)} \mid \mathbf{x}_t^{(k)}, \mathbf{z}^{(k)}\right) p\left(\mathbf{x}_t^{(k)} \mid \mathbf{y}_{1:t-1}^{(k)}, \mathbf{z}^{(k)}\right) d\mathbf{x}_t^{(k)}\right] p\left(\mathbf{z}^{(k)} \mid \mathbf{Y}^{(1:k-1)}\right) d\mathbf{z}^{(k)} \\
&= \sum_{k=1}^{K} \log \int \left[\prod_{t=1}^{T^{(k)}} \mathcal{N}\left(\mathbf{y}_t^{(k)} \mid h(\mathbf{z}^{(k)}) \boldsymbol{\mu}_{t|t-1}, h\left(\mathbf{z}^{(k)}\right) \boldsymbol{\Sigma}_{t|t-1} h\left(\mathbf{z}^{(k)}\right)^\top + \mathbf{R}\right)\right] p\left(\mathbf{z}^{(k)} \mid \mathbf{Y}^{(1:k-1)}\right) d\mathbf{z}^{(k)} \\
&= \sum_{k=1}^{K} \log \int \mathcal{N}\left(\mathrm{vec}(\mathbf{Y}^{(k)} \mid m(\mathbf{z}^{(k)}), S(\mathbf{z}^{(k)})\right) p\left(\mathbf{z}^{(k)} \mid \mathbf{Y}^{(1:k-1)}\right) d\mathbf{z}^{(k)} \\
&\approx \sum_{k=1}^{K} \log \int \mathcal{N}\left(\mathrm{vec}(\mathbf{Y}^{(k)}) \mid m(\mathbf{z}^{(k)}), S(\mathbf{z}^{(k)})\right) \mathcal{N}\left(\mathbf{z}^{(k)} \mid \boldsymbol{\mu}_{\mathbf{z}}^{(k|k-1)}, \boldsymbol{\Sigma}_{\mathbf{z}}^{(k|k-1)}\right) d\mathbf{z}^{(k)} \\
&\approx \sum_{k=1}^{K} \log \mathcal{N}\left(\mathrm{vec}(\mathbf{Y}^{(k)}) \mid m\left(\boldsymbol{\mu}_{\mathbf{z}}^{(k|k-1)}\right), \mathbf{M}\left(\boldsymbol{\mu}_{\mathbf{z}}^{(k|k-1)}\right) \boldsymbol{\Sigma}_{\mathbf{z}}^{(k|k-1)} \mathbf{M}\left(\boldsymbol{\mu}_{\mathbf{z}}^{(k|k-1)}\right)^\top + S\left(\boldsymbol{\mu}_{\mathbf{z}}^{(k|k-1)}\right)\right)
\end{aligned}
$$

where

$$
\begin{aligned}
m(\cdot) &= \mathrm{concat}\left([h(\cdot)\boldsymbol{\mu}_{1|0}, \ldots, h(\cdot)\boldsymbol{\mu}_{T^{(k)}|T^{(k)}-1}]\right) \\
S(\cdot) &= \mathrm{block\_diag}\left(h(\cdot)\boldsymbol{\Sigma}_{t|t-1} h(\cdot)^T + \mathbf{R}, \ldots, h(\cdot)\boldsymbol{\Sigma}_{t|t-1} h(\cdot)^T + \mathbf{R}\right)
\end{aligned}
$$

and $\mathbf{M}(\cdot)$ is the Jacobian of $m(\cdot)$. This block-wise approach requires materialization of the matrix $S(\cdot) \in \mathbb{R}^{NT \times NT}$, which quickly becomes intractable for large T (i.e., long trials or blocks of trials).

bioRxiv preprint doi: https://doi.org/10.64898/2026.03.07.710319; this version posted March 10, 2026. The copyright holder for this preprint (which was not certified by peer review) is the author/funder, who has granted bioRxiv a license to display the preprint in perpetuity. It is made available under aCC-BY 4.0 International license.

C.2 Augmented Space Approach

We denote the augmented state for trial k and timestep t as

$$\mathbf{u}_t^{(k)} = \begin{bmatrix} \mathbf{x}_t^{(k)} \\ \mathbf{z}^{(k)} \end{bmatrix} \in \mathbb{R}^{\frac{D(2N-D+1)}{2}} \tag{C.1}$$

where $\mathbf{x}_t^{(k)} \in \mathbb{R}^D$ and $\mathbf{z}^{(k)} \in \mathbb{R}^{\frac{D(2N-D-1)}{2}}$. In this augmented space, the within-trial dynamics is:

$$\mathbf{u}_{t+1}^{(k)} = \mathbf{A}_{\text{w.t.}}\mathbf{u}_t^{(k)} + \boldsymbol{\epsilon}_t^{(k)}, \boldsymbol{\epsilon}_t^{(k)} \sim \mathcal{N}(\mathbf{0}, \mathbf{Q}_{\text{w.t.}}), 1 \leq t < T \tag{C.2}$$

where $\mathbf{A}_{\text{w.t.}} = \begin{bmatrix} \mathbf{A} & \mathbf{0} \\ \mathbf{0} & \mathbf{I}_{\frac{D(2N-D-1)}{2}} \end{bmatrix}$, $\mathbf{Q}_{\text{w.t.}} = \begin{bmatrix} \mathbf{Q} & \mathbf{0} \\ \mathbf{0} & \mathbf{0}_{\frac{D(2N-D-1)}{2}} \end{bmatrix}$, and T is the length of a trial. At trial boundaries, we reset the latent states $\mathbf{x}_t^{(k)}$ to their initial distribution, while the displacement $\mathbf{z}^{(k)}$ evolves per their random walk dynamics, resulting in the following across-trial dynamics:

$$\mathbf{u}_1^{(k+1)} = \mathbf{A}_{\text{a.t.}}\mathbf{u}_T^{(k)} + \mathbf{b}_{\text{a.t.}} + \boldsymbol{\epsilon}_1^{(k+1)}, \boldsymbol{\epsilon}_1^{(k+1)} \sim \mathcal{N}(\mathbf{0}, \mathbf{Q}_{\text{a.t.}}) \tag{C.3}$$

where $\mathbf{A}_{\text{a.t.}} = \begin{bmatrix} \mathbf{0} & \mathbf{0} \\ \mathbf{0} & \mathbf{I}_{\frac{D(2N-D-1)}{2}} \end{bmatrix}$, $\mathbf{b}_{\text{a.t.}} = \begin{bmatrix} \boldsymbol{\mu}_{\mathbf{x}_1} \\ \mathbf{0}_{\frac{D(2N-D-1)}{2}} \end{bmatrix}$, and $\mathbf{Q}_{\text{a.t.}} = \begin{bmatrix} \boldsymbol{\Sigma}_{\mathbf{x}_1} & \mathbf{0} \\ \mathbf{0} & \text{diag}(\boldsymbol{\tau}_{\mathbf{z}}^2) \end{bmatrix}$. Finally, we can express the mapping from this space to the observations as:

$$\mathbf{y}_t^{(k)} = h_{\text{aug}}\left(\mathbf{u}_t^{(k)}\right) + \mathbf{r}_t^{(k)}, \ \mathbf{r}_t^{(k)} \sim \mathcal{N}(\mathbf{0}, \mathbf{R}) \tag{C.4}$$

bioRxiv preprint doi: https://doi.org/10.64898/2026.03.07.710319; this version posted March 10, 2026. The copyright holder for this preprint (which was not certified by peer review) is the author/funder, who has granted bioRxiv a license to display the preprint in perpetuity. It is made available under aCC-BY 4.0 International license.

h_{aug} is defined as a nonlinear transformation:

$$h_{\text{augmented}}\left(\mathbf{u}_t^{(k)}\right) = \mathbf{U}_{\text{base}} h\left(\begin{bmatrix}\mathbf{0} & \mathbf{I}_{\frac{D(2N-D-1)}{2}}\end{bmatrix}\mathbf{u}_t^{(k)}\right)\mathbf{O}_{\text{readout}}\begin{bmatrix}\mathbf{I}_D & \mathbf{0}\end{bmatrix}\mathbf{u}_t^{(k)}$$

where $\mathbf{u}_t^{(k)}$ is the augmented state at time t of trial k. $\mathbf{U}_{\text{base}}$ and $\mathbf{O}_{\text{readout}}$ are defined in Sec. 3.1. Intuitively, this function first splits the augmented state $\mathbf{u}_t^{(k)}$ into $\mathbf{z}^{(k)} = \begin{bmatrix}\mathbf{0} & \mathbf{I}_{\frac{D(2N-D-1)}{2}}\end{bmatrix}\mathbf{u}_t^{(k)}$ and $\mathbf{x}_t^{(k)} = \begin{bmatrix}\mathbf{I}_D & \mathbf{0}\end{bmatrix}\mathbf{u}_t^{(k)}$ and then applies the function $h(\cdot)$ defined in Sec. 3.1. Using this formulation, we use an EKF in the augmented state space to obtain marginal log-likelihood estimates for SMDS.

bioRxiv preprint doi: https://doi.org/10.64898/2026.03.07.710319; this version posted March 10, 2026. The copyright holder for this preprint (which was not certified by peer review) is the author/funder, who has granted bioRxiv a license to display the preprint in perpetuity. It is made available under aCC-BY 4.0 International license.

D First-order Approximation of the SMDS Emission Model

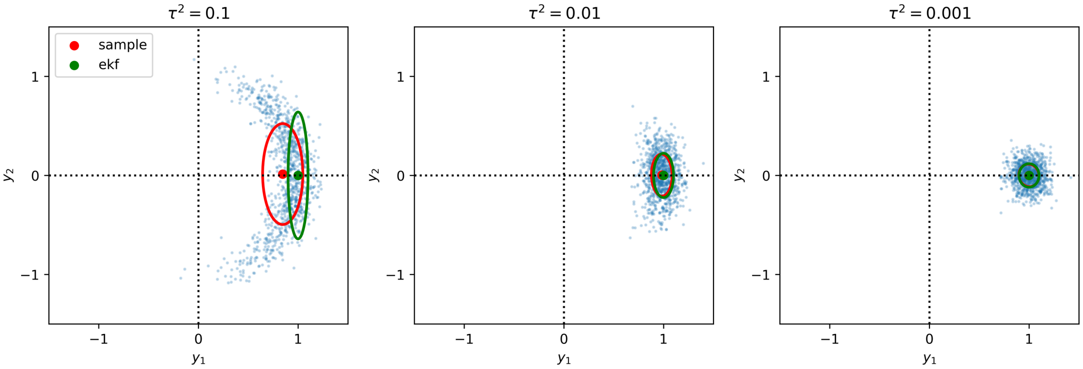

Figure 5: **First-order Taylor approximation error of the nonlinear emission model of SMDS increases as τ^2 increases.** Sample mean and covariance versus first-order Taylor approximation of the mean and covariance of the emission model of SMDS, for various levels of τ^2.

Here we explore how the extended Kalman filter and extended Kalman smoother's first-order approximation of the nonlinear emission model of SMDS changes with the drift parameter τ^2. Consider $N = 2$ and $D = 1$. Let $\mathbf{z} \sim \mathcal{N}(\mathbf{0}, \text{diag}(\tau^2))$ and $\mathbf{y} \sim \mathcal{N}(h(\mathbf{z}), \sigma^2\mathbf{I}_N)$, where $h(\cdot)$ is the nonlinear function that maps a displacement to an emission matrix, as defined in Sec. 3.1. Note that $\mathbf{z} \in \mathbb{R}^{1\times 1}$ and $\tau^2 \in \mathbb{R}^{1\times 1}_{>0}$. In addition, let $\mathbf{U}_{\text{base}} = \mathbf{I}_N$. Then, we have $h(\mathbf{z}) = f_{\mathsf{Cay}}(\mathbf{B})\mathbf{O}_{\text{readout}}$, where $\mathbf{B} = \begin{bmatrix} \mathbf{W} - \mathbf{W}^\top & \mathbf{V} \\ -\mathbf{V}^T & \mathbf{0} \end{bmatrix}$ and $\mathbf{O}_{\text{readout}} = \begin{bmatrix} \mathbf{I}_D \\ \mathbf{0} \end{bmatrix} \in \mathbb{R}^{N\times D}$. Note that for $D = 1$, $\mathbf{W}$ is a 1×1 zero matrix. For various values of τ^2, we sample 1,000 $\mathbf{V}$'s to get samples of $\mathbf{y}$'s. We compare the sample mean and covariance against their first-order approximations, which can be computed by $h(\mathbf{0})$ for the mean and $H(\mathbf{0})\text{diag}(\tau^2)H(\mathbf{0})^T + \sigma^2\mathbf{I}_N$ for the covariance, where $H(\cdot)$ is the first-order Taylor approximation of the function $h(\cdot)$. We set $\sigma^2 = 0.01$ for the visualizations

bioRxiv preprint doi: https://doi.org/10.64898/2026.03.07.710319; this version posted March 10, 2026. The copyright holder for this preprint (which was not certified by peer review) is the author/funder, who has granted bioRxiv a license to display the preprint in perpetuity. It is made available under aCC-BY 4.0 International license.

in Fig. 5. As we can see, the first-order Taylor approximation error increases as $\boldsymbol{\tau}^2$ increases. Motivated by this observation, we clip $\boldsymbol{\tau}^2$ after each M step in SMDS, such that it stays below a the clipping threshold, which is a tunable hyperparameter.

E Max $|\Delta\theta|$ (Peak Drift) Computation

Here we describe how we compute the maximum $|\Delta\theta|$. First, for each dimension d, we compute the cosine similarity for each pair of trials:

$$\cos\left(\theta_d^{(i:j)}\right) = \mathbf{C}_{:,d}^{(i)T}\mathbf{C}_{:,d}^{(j)}$$

for $i, j \in \{1, \ldots, K\}$. Here, $\mathbf{C}_{:,d}^{(k)T}$ denotes the d-th column of the emission matrix for trial k. Note that we can omit the normalization since $\mathbf{C}_{:,d}^{(k)T}$ is a unit vector. We then take the *arccos* of the cosine similarity to get $\theta_d^{(i:j)}$, the amount of rotation of the d-th dimension in radians from trial i to trial j. After converting $\theta_d^{(i:j)}$ into degrees, we compute the maximum of $\{\theta_d^{(i:j)}\}_{i,j\in\{1,\ldots,K\}}$ to get the maximum $|\Delta\theta|$ across all trials. Intuitively, this metric measures the maximum separation that ever occurred for dimension d throughout the session.

bioRxiv preprint doi: https://doi.org/10.64898/2026.03.07.710319; this version posted March 10, 2026. The copyright holder for this preprint (which was not certified by peer review) is the author/funder, who has granted bioRxiv a license to display the preprint in perpetuity. It is made available under aCC-BY 4.0 International license.

F Conditionally Linear Dynamical Systems

In the original Conditionally Linear Dynamical System (CLDS) formulation (Geadah et al., 2025), the dynamics and emission parameters vary smoothly as functions of an observed covariate $\mathbf{u}$ (e.g., heading direction or reach angle) through Gaussian process (GP) priors. To adapt CLDS for modeling representational drift, we set the covariate to the normalized trial block index, $u^{(k)} = \frac{k}{K} \in [0, 1]$, where K is the total number of blocks, and restrict non-stationarity to the emission matrix alone. Concretely, the dynamics parameters $\{\mathbf{A}, \mathbf{b}, \mathbf{Q}\}$ and the observation noise $\mathbf{R}$ are shared across all trials, while the emission matrix varies over blocks:

$$\mathbf{x}_{t+1}^{(k)} = \mathbf{A}\,\mathbf{x}_t^{(k)} + \mathbf{b} + \boldsymbol{\epsilon}_t, \tag{F.1}$$

$$\mathbf{y}_t^{(k)} = \mathbf{C}(k)\,\mathbf{x}_t^{(k)} + \boldsymbol{\omega}_t. \tag{F.2}$$

Each entry of $\mathbf{C}(k)$ is parameterized via a truncated Fourier feature expansion approximating a squared-exponential GP prior:

$$C_{ij}(k) = \sum_{\ell=1}^{L} w_\ell^{(ij)}\,\phi_\ell(k), \qquad w_\ell^{(ij)} \overset{\text{iid}}{\sim} \mathcal{N}(0, 1), \tag{F.3}$$

where $\{\phi_\ell\}_{\ell=1}^{L}$ are Fourier basis functions on a torus of period p, designed to approximate a squared-exponential GP with lengthscale κ and scale σ. Following the recommendation in the CLDS codebase[3], the period is set to $p = 1 + 6\kappa$, placing the periodic boundary well outside the covariate domain $[0, 1]$ and rendering the prior effectively non-periodic over the observed trials. The lengthscale κ governs how rapidly the emission matrix evolves across trials, playing a role analogous to the inverse of $\boldsymbol{\tau}_{\mathbf{z}}^2$ in SMDS.

[3] https://github.com/neurostatslab/clds.

bioRxiv preprint doi: https://doi.org/10.64898/2026.03.07.710319; this version posted March 10, 2026. The copyright holder for this preprint (which was not certified by peer review) is the author/funder, who has granted bioRxiv a license to display the preprint in perpetuity. It is made available under aCC-BY 4.0 International license.

Note that, unlike SMDS, CLDS does not constrain the emission matrix to be orthonormal and thus does not enforce identifiability of the latent subspace. The GP hyperparameters $\{\kappa, \sigma, L\}$ were selected via grid search over held-out log-likelihood. See Table 2 in Appendix J for the values searched.

bioRxiv preprint doi: https://doi.org/10.64898/2026.03.07.710319; this version posted March 10, 2026. The copyright holder for this preprint (which was not certified by peer review) is the author/funder, who has granted bioRxiv a license to display the preprint in perpetuity. It is made available under aCC-BY 4.0 International license.

G Simulated Data Experiments

We provide details on our simulated data experiments below. For further information on hyperparameter selection, runtime, and computational resources used in the experiments, please refer to Sec. J.

G.1 Toy Experiment

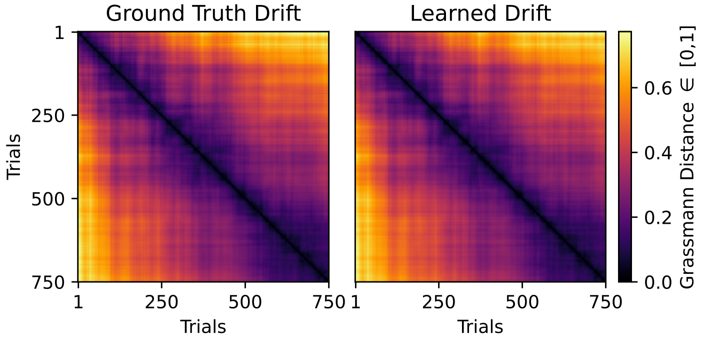

Figure 6: **Simulated and learned drift for the toy experiment in Sec. 4.1**: We simulated smooth drift and computed the normalized Grassmann distance matrix of the simulated emission matrices (left). We then fitted an SMDS and computed the normalized Grassmann distance matrix with the learned emission matrices. SMDS accurately recovers the simulated drift.

We set the ground truth latent dimensionality to $D = 2$ and the observation dimension to $N = 10$. We set the number of trial conditions to 4. For each condition, we sampled the initial mean of the latent states from a Gaussian distribution with zero mean and diagonal covariance with diagonal values set to $\sqrt{N/D}$. The dynamics matrix was set to a random rotation matrix, and the dynamics and emissions noises were set to 0.01. To generate drift, we sampled displacements $\{\mathbf{z}^{(k)}\}_{k=1}^{K}$ for $K = 750$ trials from a random walk, where the drift rates were sampled independently per dimension: $\tau_i^2 \in [10^{-10}, 10^{-4}]$, $i \in \{1, \dots, \frac{D(2N-D-1)}{2}\}$. The drift rate τ^2 for the true model was then set to the empirical variance of the smoothed displacements. A total of 125 trials were left out for test

bioRxiv preprint doi: https://doi.org/10.64898/2026.03.07.710319; this version posted March 10, 2026. The copyright holder for this preprint (which was not certified by peer review) is the author/funder, who has granted bioRxiv a license to display the preprint in perpetuity. It is made available under aCC-BY 4.0 International license.

data. We fitted both models for 200 EM iterations. Fig. 6 shows the normalized Grassmann distance matrix computed with the simulated emission matrices (left) and with the learned emission matrices (right). SMDS accurately recovers the ground truth drift.

G.2 Higher-dimensional Simulated Data Experiment

We validated whether SMDS could recover latent dynamics and observation drift in higher-dimensional setups. We simulated data from an SMDS with $D = 8$ and $N = 24$. We fitted both LDS and SMDS across a range of state dimensions, $D \in [4, 16]$. As shown in Fig. 7A, SMDS accurately identified the true latent dimensionality of 8, while we once again observed that the test log-likelihood of LDS continued to increase beyond the true dimension and underperformed relative to SMDS. We also found that SMDS accurately recovered the eigenvalues of the true dynamics matrix (Fig. 7B), whereas LDS failed to do so.

To assess the ability of SMDS to recover drift, we computed pairwise Grassmann distances (described in Sec. 2.3) using the ground-truth and learned emission matrices between trials (Fig. 7C). We found that SMDS accurately captured the drift pattern in the ground-truth data. We also evaluated whether SMDS could recover the relationship between peak drift (defined in Appendix E) and explained variance across individual subspace dimensions. We made this relationship identifiable by rotating the emission matrices to order the dimensions by their average explained variance. As shown in Fig. 7D, SMDS recovered the ground-truth trend accurately.

These experiments demonstrate the ability of SMDS to accurately capture latent dynamics in the presence of drift. SMDS recovers the correct latent dimensionality and obtains

bioRxiv preprint doi: https://doi.org/10.64898/2026.03.07.710319; this version posted March 10, 2026. The copyright holder for this preprint (which was not certified by peer review) is the author/funder, who has granted bioRxiv a license to display the preprint in perpetuity. It is made available under aCC-BY 4.0 International license.

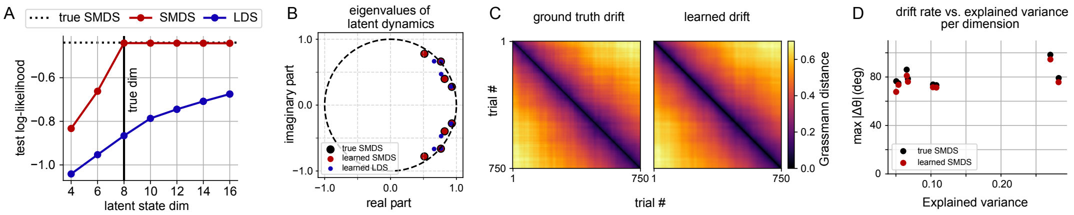

Figure 7: **Simulated data experiment 2.** **(A)**: Held-out log-likelihood on data simulated from SMDS with $D = 8$, $N = 24$. SMDS outperforms standard LDS and recovers the true state dimension. **(B)**: Eigenvalues of the true dynamics matrix, A, compared to those learned by SMDS and LDS. SMDS recovers these accurately. **(C)**: SMDS also recovers ground truth drift, measured by the normalized Grassmann distance across 750 trials. **(D)**: We show the relationship between peak drift and explained variance across individual subspace dimensions from the ground truth data. SMDS accurately recovers this relationship.

a higher held-out test log-likelihood than an LDS. LDS requires more latent dimensions for the same dataset, highlighting the need to account for drift. SMDS also allows for precise quantification of drift in the observation space.

G.3 Experiments with Data Simulated from an LDS

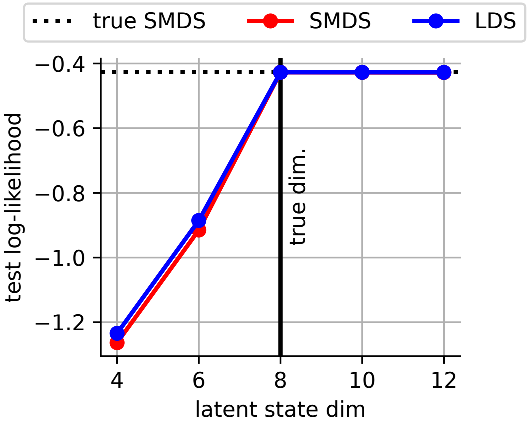

Figure 8: **Test log-likelihood plot for the LDS simulated experiment in Sec. 4.1**: We simulated data from an LDS with $D = 8$ and $N = 24$. We fitted both LDS and SMDS with state dimensions ranging from 4 to 12.

As a control, we simulated stationary data from an LDS ($D = 8$, $N = 24$) and fitted

bioRxiv preprint doi: https://doi.org/10.64898/2026.03.07.710319; this version posted March 10, 2026. The copyright holder for this preprint (which was not certified by peer review) is the author/funder, who has granted bioRxiv a license to display the preprint in perpetuity. It is made available under aCC-BY 4.0 International license.

both LDS and SMDS. The emission matrix was set randomly with a standard normal distribution, and the dynamics matrix was set to a random rotation matrix. The dynamics and emissions noises were set to 0.1. We sampled a total of 750 trials, each of which was 30 timesteps long. We fitted SMDS and LDS with state dimensions ranging from 4 to 12. For SMDS, we set the α and β for the prior on $\boldsymbol{\tau}^2$ to 1e2 and 1e−9, respectively, and the initial value of $\boldsymbol{\tau}^2$ to 1e−9. The clipping value of $\boldsymbol{\tau}^2$ was set to 1e−4. Both models achieved peak test log-likelihood at the true latent dimensionality. LDS slightly outperformed SMDS due to the additional flexibility in modeling displacement dynamics for stationary data. This further validates our inference procedure and demonstrates that SMDS appropriately captures stationary datasets.

bioRxiv preprint doi: https://doi.org/10.64898/2026.03.07.710319; this version posted March 10, 2026. The copyright holder for this preprint (which was not certified by peer review) is the author/funder, who has granted bioRxiv a license to display the preprint in perpetuity. It is made available under aCC-BY 4.0 International license.

H Modeling Macaque Neural Data

We include further details for the macaque neural data example presented in Sec. 4.2. For further information on hyperparameter selection, runtime, and computational resources used in the experiments, please refer to Sec. J.

H.1 Training Details and Hyperparameters

We chunked the trials into blocks of 8 trials, resulting in a total of 93 blocks. Keeping the 8 blocks at each end of the session as training data, we randomly sampled 12 blocks from the remaining blocks for test data across 5 different seeds. To prevent unstable inference caused by overfitting to some channels, we clipped the entries of the diagonal emission covariance matrix to a minimum of either $5e{-3}$ or $4e{-3}$. We fitted both models for 300 EM iterations, and saw that the training log-likelihood saturated.

I Modeling Rodent Neural Data

We include further details for the rodent neural data example presented in Sec. 4.3. For further information on hyperparameter selection, runtime, and computational resources used in the experiments, please refer to Sec. J.

I.1 Training Details and Hyperparameters

We chunked the trials into blocks of 4 trials, resulting in a total of 31 blocks. Keeping the 6 blocks at each end of the session as training data, we randomly sampled 3 blocks from the remaining blocks for test data across 3 different seeds. We fitted both models for 600 EM iterations, and ensured that the training log-likelihood saturated.

bioRxiv preprint doi: https://doi.org/10.64898/2026.03.07.710319; this version posted March 10, 2026. The copyright holder for this preprint (which was not certified by peer review) is the author/funder, who has granted bioRxiv a license to display the preprint in perpetuity. It is made available under aCC-BY 4.0 International license.

I.2 PCA Cumulative Explained Variance

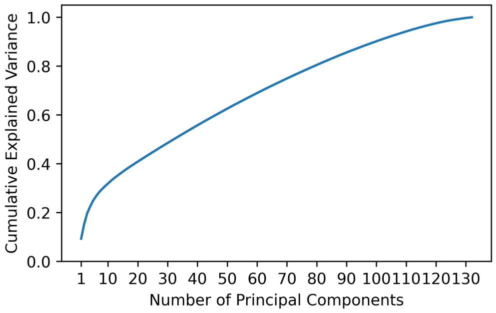

Figure 9: Cumulative explained variance as a function of number of principal components.

We show the PCA cumulative explained variance plot as a function of the number of principal components (Fig. I). We see a kink at around 10 principal components, hence we fit SMDS and LDS with state dimensions from 1 to 10 in our main experiment in Sec. 4.3.

bioRxiv preprint doi: https://doi.org/10.64898/2026.03.07.710319; this version posted March 10, 2026. The copyright holder for this preprint (which was not certified by peer review) is the author/funder, who has granted bioRxiv a license to display the preprint in perpetuity. It is made available under aCC-BY 4.0 International license.

J Experimental Configurations

Table 1: Outline of the key hyperparameters in LDS and SMDS.

Hyperparameter	Description	Applicable Models	Permissible Values
D	Dimension of the latent state space.	LDS, SMDS, CLDS	$\mathbb{Z}_{\geq 1}$
α	Shape of the Inverse-gamma prior on $\boldsymbol{\tau}^2$.	SMDS	$\mathbb{R}_{>0}$
β	Scale of the Inverse-gamma prior on $\boldsymbol{\tau}^2$.	SMDS	$\mathbb{R}_{>0}$
Initial $\boldsymbol{\tau}^2$	Initialization value of $\boldsymbol{\tau}^2$.	SMDS	$\mathbb{R}_{>0}$
Clipping value of $\boldsymbol{\tau}^2$	An upper limit on $\boldsymbol{\tau}^2$	SMDS	$\mathbb{R}_{>0}$
κ	GP lengthscale	CLDS	$\mathbb{R}_{>0}$
σ	GP scale	CLDS	$\mathbb{R}_{>0}$
L	Number of basis functions for GP approximation	CLDS	$\mathbb{Z}_{\geq 1}$

Unless otherwise specified, we performed a grid search over a range of values within the permissible set. In certain circumstances, the hyperparameter was selected to consider empirical properties of the data, e.g. we used D from 1 to 10 for the rodent neural data based on our observation in Section 9. In addition, for fair comparison, we initialized both $\mathbf{U}_{\text{base}}$ of SMDS and the emission matrix of LDS with PCA. Below is a table that lists the hyperparameter search details for Sec. 4.1, 4.2, and 4.3.

Table 2: Experiment-specific Hyperparameter Search.

Hyperparameter	Sim. Exp. 1 (Sec. 4.1)	Sim. Exp. 2 (Sec. 4.1)	Macaque Data (Sec. 4.2)	Rodent Data (Sec. 4.3)
D (LDS)	$\{1, 2, \ldots, 10\}$	$\{4, 6, \ldots, 16\}$	$\{5, 10, \ldots, 35\}$	$\{1, 2, \ldots, 10\}$
D (SMDS)	$\{1, 2, 3, 4\}$	$\{4, 6, \ldots, 12\}$	$\{5, 10, \ldots, 35\}$	$\{1, 2, \ldots, 10\}$
D (CLDS)	N/A	N/A	$\{5, 10, \ldots, 35\}$	$\{1, 2, \ldots, 10\}$
α	1e−6	{1e0, 5e0, 7e0, 1e1, 2e1, 3e1, 5e1}	{1e−3, 1e−2, 1e−1}	{1e−6, 1e−5, 1e−3}
β	1e−6	1e−9	1e−6	{1e−5, 1e−4}
Initial $\boldsymbol{\tau}^2$	1e−6	{1e−8, 5e−8, 1e−7, 5e−7, 1e−6}	{5e−6, 7e−6, 1e−5, 2e−5}	{5e−7, 1e−6, 5e−6, 1e−5}
Clipping value of $\boldsymbol{\tau}^2$	1e−3	1e−4	1e−4	1e−4
κ	N/A	N/A	{0.4, 0.45, 0.5}	{0.4, 0.45, 0.5}
σ	N/A	N/A	0.2	0.2
L	N/A	N/A	{5, 9, 13, 17, 65, 71, 77, 83}	{5, 9, 13, 17, 65, 71, 77, 83}

bioRxiv preprint doi: https://doi.org/10.64898/2026.03.07.710319; this version posted March 10, 2026. The copyright holder for this preprint (which was not certified by peer review) is the author/funder, who has granted bioRxiv a license to display the preprint in perpetuity. It is made available under aCC-BY 4.0 International license.

K PCA Fails to Recover Drift Per Dimension

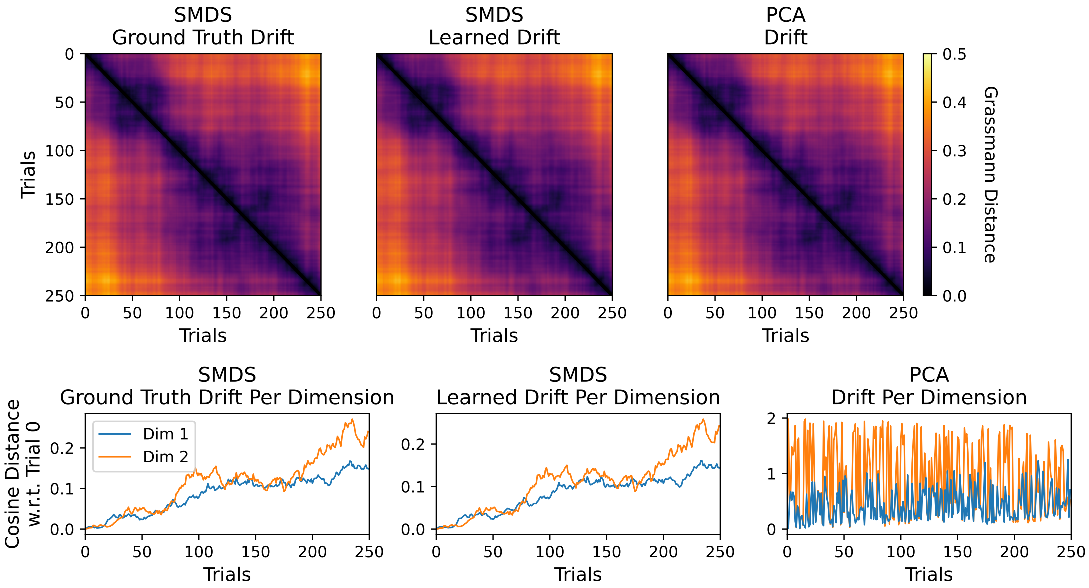

Figure 10: **PCA fails to recover per-dimension drift. (Top Row):** Ground-truth drift from SMDS (left), drift estimated by SMDS (center), and drift estimated by PCA (right). Both SMDS and PCA are able to recover the true overall drift measured by the normalized Grassmann distance. **(Bottom Row):** Drift per dimension from true SMDS (left), learned SMDS (center), and PCA (right). While SMDS accurately recovers the true drift per dimension, measured by cosine distance relative to the first trial, PCA does not.

We set the ground truth latent dimensionality to $D = 2$ and the observation dimension to $N = 10$. The dynamics matrix was set to a random rotation matrix, and the dynamics and emissions noises were set to 0.01. To generate smooth drift, we first sampled displacements $\{\mathbf{z}^{(k)}\}_{k=1}^{K}$ for $K = 250$ trials from a random walk, where the drift rates were sampled independently per dimension: $\boldsymbol{\tau}_i^2 \in [10^{-10}, 10^{-4}]$, $i \in \{1, \ldots, \frac{D(2N-D-1)}{2}\}$. The drift rate $\boldsymbol{\tau}^2$ for the true model was then set to the empirical variance of the smoothed displacements. Each trial was 200 timesteps long.

As shown in Figure 10, SMDS was able to recover both the overall drift and the drift

bioRxiv preprint doi: https://doi.org/10.64898/2026.03.07.710319; this version posted March 10, 2026. The copyright holder for this preprint (which was not certified by peer review) is the author/funder, who has granted bioRxiv a license to display the preprint in perpetuity. It is made available under aCC-BY 4.0 International license.

per individual dimension. On the other hand, PCA accurately captured the overall amount of drift but failed to track how individual dimensions drifted over trials. This shows the failure modes of PCA: the principal components can flip signs or swap their relative ordering between trials, preventing PCA from recovering the true per-dimension drift.

bioRxiv preprint doi: https://doi.org/10.64898/2026.03.07.710319; this version posted March 10, 2026. The copyright holder for this preprint (which was not certified by peer review) is the author/funder, who has granted bioRxiv a license to display the preprint in perpetuity. It is made available under aCC-BY 4.0 International license.

L Code Availability

Our implementation of SMDS is available at `https://github.com/lindermanlab/smds`. The repository includes the source code for SMDS (in Python) and an example Jupyter Notebook. This notebook shows how to sample data from an SMDS and then re-fit an SMDS with the procedure described in Sec. 3.2. SMDS is built based on the framework provided by Dynamax Linderman et al. (2025).
